# Potential therapeutic targets in the prevention of testicular ischemia-reperfusion injury

**DOI:** 10.3389/frph.2025.1706914

**Published:** 2025-11-18

**Authors:** Richard Adedamola Ajike, Ayodeji Folorunsho Ajayi, Olubunmi Simeon Oyekunle, Waidi Adeoye Saka, Sodiq Opeyemi Hammed, Oreoluwa Janet Adedeji, Olajumoke Deborah Ogunleye, Oluwaseun Samuel Hezekiah, Oluwakemi Victoria Olayinka-Akinpelu, Babatunde Adebola Alabi, Ishola David Ajao, Oladele Ayobami Afolabi

**Affiliations:** 1 College of Health Science, LAUTECH, Ogbomoso, Nigeria; 2 Biology Department, Trinity Christian College, Palos Heights, IL, United States; 3 Pan African Cancer Research Institute, University of Pretoria, Prinshof Campus, Pretoria, South Africa; 4 Department of Nursing, Kings University, Ode Omu, Nigeria

**Keywords:** testicular ischemia/reperfusion injury, testicular torsion, pharmacological postconditioning, oxidative stress, inflammation

## Abstract

Testicular ischemia-reperfusion injury (TIRI) is the outcome of the repair of torsion of the testis. It has been reported to cause loss of testicular function in both the ipsilateral and contralateral testes in the long run, thus resulting in male infertility. Its prevention is complex due to activation of oxidative stress, inflammation and apoptotic pathways in the ischemic and reperfusion phases. Previous experimental studies have successfully mitigated TIRI by applying ischemic preconditioning, ischemic postconditioning and pre-treatment regimens, which may not be appropriate for humans due to limitations associated with their application in real-life situations. However, pharmacological postconditioning, which involves the use of drugs to block key points in the TIRI pathway, can be proactively applied in humans, offering a better TIRI management strategy. Pathophysiological events in the TIRI pathway include activation of: xanthine oxidase (XO)-reactive oxygen species (ROS) pathway in the ischemic phase, calcium-mediated apoptotic pathway in the early reperfusion phase, and ROS-burst in the late reperfusion phase, among others. Hence, this review recommends that blocking the XO-ROS pathway with febuxostat after the onset of testicular torsion (TT), minimizing the calcium-mediated apoptotic pathway and restoring the loss of vasomotor tone with amlodipine on reperfusion, as well as blocking ROS-burst with vitamin E in the later phase of reperfusion, may help to mitigate the effect of TIRI in humans and thus prevent future infertility. Nevertheless, further research is needed to verify this claim and delineate the possible drug-drug interactions, as well as potential effects on other organs.

## 1 Testicular torsion and repair: a dilemma akin to jumping out of the frying pan into the fire

Testicular Torsion (TT) is an emergency condition characterized by the twisting of the spermatic cord and its content (1, 2). It has an annual incidence of about 1 in 4,000 males (3, 4). Previous studies have reported variation in the incidence of TT from continent to continent, with Sub-Saharan African, South American and North American regions reporting annual incidences of 2.7, 1.4 and 3.8, respectively, per 100,000 men below the age of 40 (5). Although TT affects males of all ages (6), the common age of occurrence is between 12 and 18 years, even up to 24 years and above (7). Symptoms of TT include: severe scrotal pain, testicular swelling and reddening of the scrotal skin (8, 9). Previous studies have also documented that TT contributed about 1.8% to male infertility in Africa (7, 10, 11). Epidemiological studies further conducted in North-Eastern Nigeria reported that TT accounts for about 5.8% of testicular insufficiency among other causes (12). Some predisposing factors to TT include bell clapper deformity (an anatomical abnormality present in about 12% of males), increased testicular volume (at the onset of puberty), testicular trauma (seen in accidents that cause impact on the scrotum), intense sporting activities such as cycling, weight lifting, testicular injuries during football or baseball, cryptorchidism, hyperactivity of cremasteric muscle and during sleep without any prior trauma (8, 13–18).

In clinical practice, when TT occurs, the main management stratagem is surgical detorsion (SD), which has to be done quickly to re-establish blood flow and prevent necrosis, as well as ease the ischemic pain experienced (19, 20). While important, SD should be regarded as a “necessary evil”. Because, despite being done to prevent necrosis, testicular ischemia-reperfusion injury (TIRI) still occurs (21), which exacerbates testicular damage, causes late organ damage, and infertility in the long run through oxidative stress, inflammation and apoptosis (22–26).

**Figure 1:**
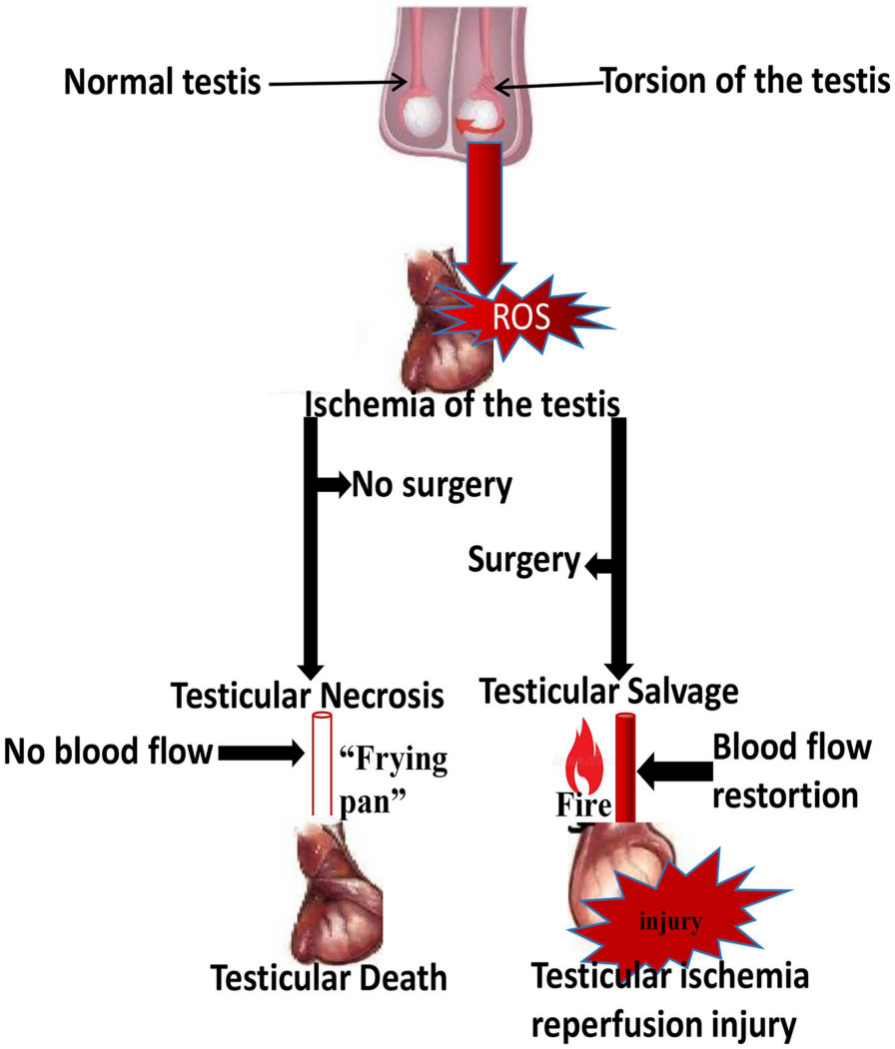
Diagram illustrating the pathophysiological outcomes of testicular torsion and subsequent detorsion.

TIRI poses a serious risk in the long run, involving the later loss of both the ipsilateral and contralateral testes, thereby resulting in permanent infertility (27–29). Notably, this risk might even be higher compared to the risk of losing only the ipsilateral testes to necrosis when not repaired (30). Hence, both conditions present as dilemmas, which can be tagged by the phrase “jumping out of the frying pan into the fire”. The frying pan, in this case, is the torsed testes becoming necrosed, with no other option than to carry out orchiectomy, while the fire is the outcome of TT repair. TIRI has been reported to affect male reproductive capacity by causing: degeneration of seminiferous tubules (31–34), disruption of Sertoli cell protein (31, 35), alteration of reproductive hormone production (36–38), loss of vasomotor tone (32), testicular atrophy and ultimately infertility (2, 39, 40). The consequences of testicular torsion onset and its repair are illustrated below (Figure 1).

Testicular torsion interrupts blood flow, resulting in ischemia and reactive oxygen species (ROS) generation. In the absence of surgical intervention (“frying pan”), prolonged ischemia causes testicular necrosis and eventual testicular death. Surgical detorsion (“fire”) restores blood flow but triggers an oxidative burst and inflammatory response, leading to testicular ischemia-reperfusion injury (TIRI). The analogy of “from the frying pan into the fire” depicts the paradox wherein surgical correction prevents necrosis yet induces reperfusion-mediated oxidative damage.

## 2 Overview of testicular ischemia-reperfusion injury (TIRI) and the complexity involved in its prevention

Testicular ischemia-reperfusion injury (TIRI) generally involves two phases: the ischemic and reperfusion phases (25). The ischemic phase results in the build-up of xanthine oxidase (XO) enzymes, a radical reactive oxygen species (ROS) generator that may cause early organ damage (24, 41). This phase is also accompanied by depletion of oxygen and nutrient supplies, build-up of free radicals leading to increased oxidative stress (42), decreased intracellular ATP, glycogen and calcium overload due to damage to the ATPase pump (39). The second phase is the reperfusion phase, and it is characterized by the activation of inflammatory and calcium-induced apoptotic pathways and production of peroxynitrite radicals, which interfere with cellular structures such as proteins, lipids and DNA and further cause severe oxidative damage to the testes (22–24).

**Figure 2:**
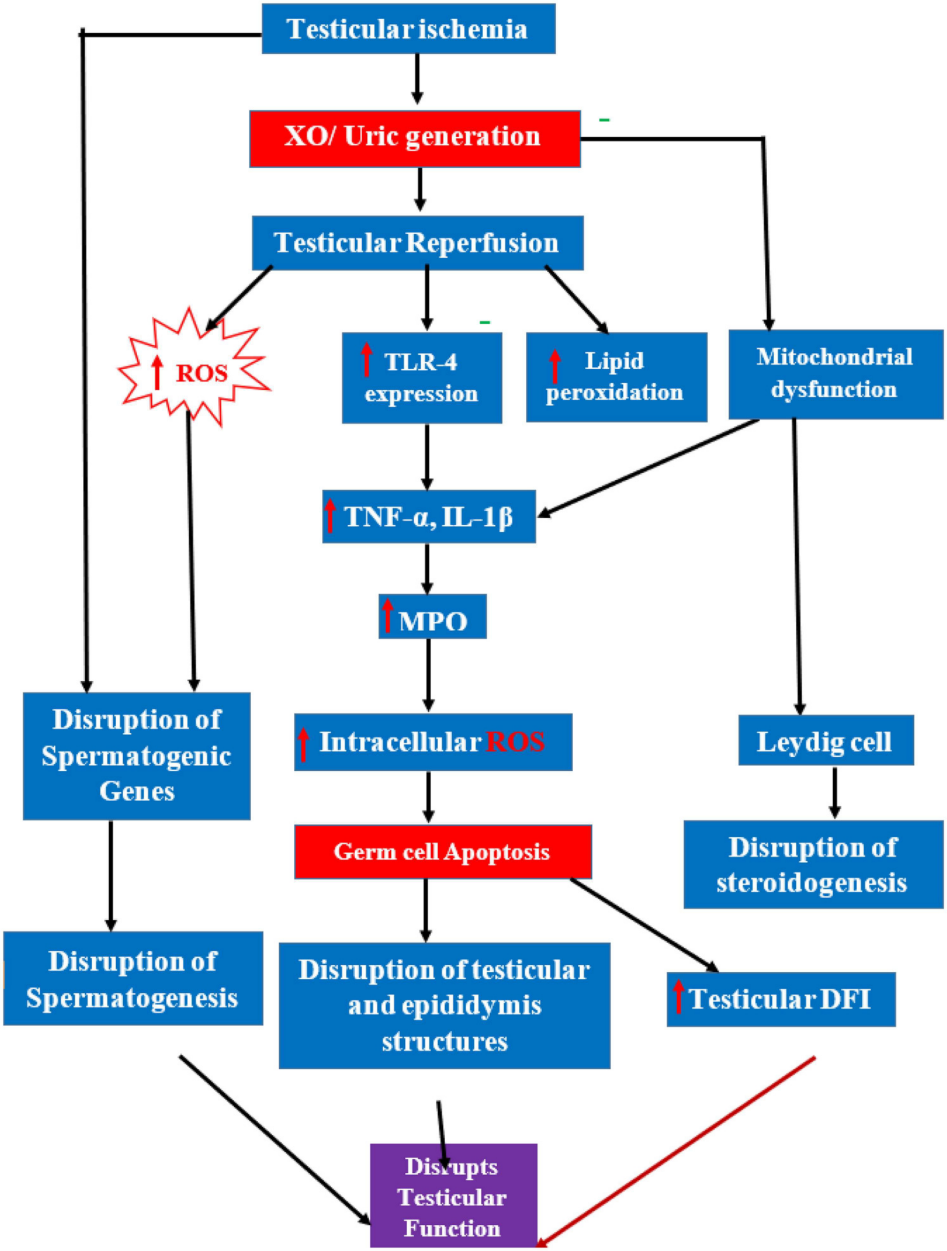
Diagram illustrating cascades of events activated during testicular ischemia-reperfusion injury.

The prevention of TIRI may be complex for the following reasons. The first is that the two phases involved contribute to testicular damage together. For instance, there is a build-up of xanthine oxidase (XO) enzymes in the ischemic phase that trigger reactive oxygen species (ROS) production that can inflict immediate oxidative injury to the testicular tissue (20, 43). The ROS produced in the ischemic phase also form complexes with other molecules and proteins to cause massive damage to the testes during reperfusion. The XO-ROS production in the ischemic phase also serves as a basis for the formation of peroxynitrite complexes that can cause long-term damage to the testicular tissue throughout the entire period of reperfusion (44). Secondly, other pathophysiological pathways are activated during the reperfusion phase (25, 27, 28, 126), which have to be addressed urgently. These events include the disruption of the sodium-calcium ATPase pump, which results in excess calcium influx into the cell to initiate the calcium-mediated apoptotic pathway that triggers germ cell loss (45–47). There is also loss of vasomotor tone after torsion repair, which does not return to normal until after 7 days of reperfusion (48). This, of no doubt, disrupts blood flow to the testes, thereby affecting spermatogenesis. Furthermore, the events of reperfusion include an increased inflammatory response. This is typically due to the activation of toll-like receptors that increase the release of tumor necrosis factor alpha and interleukin-1-beta from the macrophages and dendritic cells into the blood (49–51, 127). These cytokines facilitate the mobilization of neutrophils to the site of injury and feed back into different ROS pathways to increase their production. Consequently, this storm of activated cytokines and ROS trigger the intrinsic and extrinsic apoptotic pathways (32, 52). This effect is referred to as ROS-burst, which occurs in the later phase of reperfusion (46, 47). The pathway in Figure 2 denotes the pathway of TIRI-induced testicular damage.

Hence, having identified different points on the TIRI pathway which contribute significantly to testicular damage, there is a need to investigate therapeutic drugs best suited to block these key points in the ischemic and reperfusion phases (53, 54). This may require the administration of the drugs in a sequence or pattern, such that damage in each phase is targeted. That is, the administration of a therapeutic drug in the ischemic phase and in the early reperfusion phase to prevent necrosis, as delayed or inappropriate management may result in the loss of testicular cells and function (55, 56).

The initial ischemic event leads to the generation of uric acid via xanthine oxidase (XO). Subsequent reperfusion triggers a significant burst of reactive oxygen species (ROS), which initiates parallel damaging cascades. These include the upregulation of Toll-like receptor 4 (TLR-4) and lipid peroxidation, leading to the release of pro-inflammatory cytokines (TNF-α, IL-1β) and subsequent myeloperoxidase (MPO)-mediated intracellular ROS production. This inflammatory cascade, along with direct ROS-induced damage to spermatogenic genes and mitochondrial dysfunction affecting Leydig cells, culminates in germ cell apoptosis, disruption of steroidogenesis, increased DNA fragmentation index (DFI), and damage to testicular structures, ultimately leading to global testicular dysfunction.

## 3 Recommended testicular ischemia-reperfusion injury (TIRI) prevention strategies

Due to the risk of future infertility associated with testicular ischemia-reperfusion injury (24, 128), studies have recommended four general management options, which are: ischemic preconditioning, pretreatment, ischemic postconditioning and pharmacological postconditioning (57).

### 3.1 Ischemic preconditioning

Ischemic preconditioning is a process whereby a short period of ischemia and reperfusion is induced in a tissue to make the tissue resistant against the ischemic insult that is about to occur (57). It confers protection against tissue damage, most especially when ischemic insult is predictable. Murry et al. (58) and Valen and Vaage (129) reported that the essence of ischemic preconditioning is to enable mammals to adapt to the ischemic insult, thereby reducing infarct size and limiting the severity of ischemia-reperfusion injury. Ischemia preconditioning has been tested in diverse organs in the literature, but conflicting results were observed among various tissues in animal models based on the cycle of ischemia and reperfusion that was induced before the exact ischemia insult or stress. Studies have reported the protective role of ischemia preconditioning protocol in the heart, liver, and kidney (130–132). In other studies, the efficacy of ischemia preconditioning has been established in cardiac surgery and percutaneous coronary interventions in humans (133). Under the ischemia-preconditioning protocol, it was observed that the protective role of ischemia is biphasic. In the first phase, the window of protection lasts less than 2 h before the sustained ischemia insult, while the second phase is the second window of protection (SWOP), which occurs 24–72 h after the sustained ischemia insult (129). Apart from the fact that ischemic preconditioning can be induced locally to monitor the local function of a particular organ, it can also be induced to observe its effect on distant organ which is referred to as remote preconditioning or by pharmacological treatment before initial ischemia known as pharmacological preconditioning (131, 134–136). Studies have shown that ischemia preconditioning is capable of improving distant organ function and local function of preconditioned organs (137–139). Ambros et al. (140) further explained that ischemia preconditioning utilized both local and distant mechanisms in the brain, skeletal muscle, liver, lungs, kidney and intestine in animal models to protect against ischemia-reperfusion injury. However, despite the reported application in its use to prevent local and distant damage in other organs, it is not appropriate for the treatment of testicular ischemia-reperfusion injury due of the unpredictability in the occurrence of TT (59, 60). Ischemia preconditioning is only effective in organ transplants such as the kidney and the heart, where ischemia reperfusion injury is predictable, unlike the case of torsion of the testes, which is unpredictable.

### 3.2 Pretreatment before the induction of testicular ischemia-reperfusion injury

This concept involves the application of various pharmacological agents before the induction of testicular torsion (TT). Ischemia preconditioning entails a brief cycle of ischemia and reperfusion to make the tissue resistant against damage before the occurrence of the real ischemic insult (57). The pretreatment strategy requires administration of various agents that possess antioxidant, anti-inflammatory and anti-apoptotic properties, as well as calcium channel blockers, before the occurrence or onset of testicular torsion. The essence of this strategy is to confer protection or neutralize the activities of any impending stress to the testicular tissue before the onset of testicular torsion. Even though, several experimental studies have reported the protective effects of antioxidant pretreatments with superoxide dismutase, catalase, melatonin and imvastatin against testicular ischemia-reperfusion injury (61, 141, 142), yet this strategy is perceived unpopular among the clinicians for the treatment of TT in humans due to unpredictability in the occurrence of TT (3). This option could only serve as a prophylactic measure in tissues where the onset of tissue injury, such as kidney and liver transplants, is predictable, unlike the case of TT onset in humans, which is unpredictable. Based on this, its application in the treatment of TT in real-life situations is unrealistic. Thus, it cannot be applied for the treatment of TT in humans. In addition to this, because the exact time of onset of TT is not known, the application of this strategy may not be effective in preventing the activities of debilitating factors that accompany the onset of TT.

### 3.3 Ischemic postconditioning

Ischemic postconditioning is a protective strategy employed to reduce the injury caused by ischemia (obstruction in blood flow) and reperfusion (restoration of blood flow) in tissues. This technique has been applied primarily in the cases of heart attacks or cardiac surgery. It also involves induction of a brief cycle of ischemia and reperfusion at the start of reperfusion after sustained ischemia stress (143, 144). It also involves intermittent obstruction of blood flow at the early phase of reperfusion to reduce the risk of reperfusion injury (57). This protocol has been reported to reduce infarct size and is more effective than ischemic preconditioning (143, 145). The concept of ischemic postconditioning as a management strategy after cardiac tissue transplant showed that induction of a brief cycle of coronary ischemia for 30 s, followed by reperfusion for 30 s for three consecutive cycles at the onset of reperfusion, reduced the size of the infarction. Skyschally et al. (146) also reported that factors such as delay after the first re-occlusion is established, duration and number of re-occlusions and duration of interspersed reperfusion must be considered for ischemic postconditioning to be effective.

Another controversial aspect of ischemic postconditioning is targeting the exact time the brief cycle of ischemia and reperfusion must be done at the onset of reperfusion to avoid delay of any kind. Studies have documented that ischemia postconditioning may still reduce infarct size when there is a delay to the first re-occlusion within 1–3 min, while other studies reported that delay had no impact on reducing infarction size when ischemia postconditioning was initiated at the onset of reperfusion (144, 147, 148). However, the long-term cycle of ischemia and reperfusion at the onset of reperfusion does not prevent the damaging effect of ischemia-reperfusion injury (144, 146). Thereby, suggesting the significance of the brief cycle of occlusion and re-occlusion of the vessels after the onset of reperfusion and also, it seemed that the potency of this protocol in reducing infarct size is dependent on establishing the protocol at the right or specific time during the onset of reperfusion.

The application of ischemic postconditioning in the management of testicular ischemia-reperfusion injury in humans is perceived to be less important because it does not account for or take care of certain events that occur in the ischemic phase, which could inflict injury on testicular cells. Also, this protocol may result in damage to the testicular artery when practiced on humans due to the need for occlusion and re-occlusion of the vessels at the onset of reperfusion several times. This protocol may be accompanied by complications that may exacerbate testicular damage. Based on the emergency nature of testicular torsion and the urgent need for treatment, the basis of ischemic post-conditioning may not have been effective for the management of TIRI in humans.

### 3.4 Pharmacological postconditioning

Pharmacological postconditioning refers to the use of pharmacological agents to mimic or enhance the effects of ischemic postconditioning, which helps protect tissues from ischemia-reperfusion injury (IRI) (62). Unlike ischemic postconditioning, which involves physical interventions like brief cycles of blood flow restriction after reperfusion, pharmacological postconditioning uses specific drugs to activate cellular protective pathways that reduce injury caused by the restoration of blood flow after ischemia (62).

Pharmacological postconditioning (PPC) involves the application of drugs during the period of ischemia or at the onset of reperfusion to block key points on the ischemia-reperfusion injury pathway (63, 149). In an attempt to mitigate the effect of TIRI in experimental studies, conventional prevention strategies, including the use of anti-inflammatory medications, apoptosis inhibitors, and antioxidants, have been applied (64, 65), but their effectiveness is hindered by significant limitations (66). Though there were evidences of improvement in testicular function, these conventional prevention strategies do not properly take care of the multifactorial nature of the TIRI pathway. The multifactorial nature supports the need for the use of two or more therapeutic drugs to block key points on the TIRI pathways. Due to these aforementioned factors, pharmacological postconditioning may be effective for the management of TIRI due to the practicability in its use for the treatment of TIRI in humans, less risk associated with its application and possibilities of increasing the chances of treatment by blocking key points on the TIRI pathways through the use of different therapeutic drugs (63). PPC exhibited remarkable superiority in reducing inflammation and oxidative stress (67), inhibiting apoptosis (68), and enhancing endothelial function (69). Moreover, PPC has consistently demonstrated its ability to minimize testicular damage and improve fertility in animal models by inhibiting key pathways of inflammation, apoptosis, and oxidative stress (60, 66, 67).

Although, this concept is not currently in use in clinical practice for the management of TIRI in humans, reports on its effectiveness in minimizing TIRI in rats have been established (57, 62). Therefore, investigating the application of PPC in experimental studies may help to improve the treatment of TIRI in humans. The multifaceted benefits of PPC make it an attractive alternative to traditional preventive strategies.

## 4 Unreliability in the use of ischemic preconditioning, postconditioning and pretreatment regimen in the treatment of testicular ischemia-reperfusion injury

The use of ischemic preconditioning, postconditioning and pre-treatment regimen for the treatment of testicular ischemia-reperfusion injury (TIRI) remains largely impractical in humans. Exposing tissue to short periods of ischemia and immediate reperfusion before the onset of ischemia (ischemic preconditioning) renders the tissue flexible against the impending ischemic events (70). The use of ischemic preconditioning strategy helps the tissue to develop a balance between free radicals generated and tissue defense system to prevent oxidative stress and inflammation to prevent IRI (71). This protocol is only effective in pre-arranged procedures like cardiac surgery, where the occurrence of ischemia is predictable, but not in the case of torsion of the testes, where the onset of ischemia is not predictable. Therefore, the unpredictability in the occurrence of testicular torsion in humans, which results in testicular ischemia, makes ischemic preconditioning totally unreliable in managing TIRI following a successful detorsion (72). In tissue like the heart, ischemic preconditioning helps to significantly reduce necrosis by about 30%–40% (73), but in tissue that constantly undergoes aerobic metabolism like the testis, a little period of ischemia adversely triggers the production of free radicals, oxidative stress and ROS-induced apoptosis (74), causing the testis to experience cessation of spermatogenesis (the primary function of the testis) even with a short period of ischemia (17). Application of ischemic preconditioning on the testis may deprive testicular cells, especially the Sertoli and Leydig cells, of oxygen and required nutrients, causing abnormal sperm qualities or total cessation of spermatogenesis (75).

In addition to this, exposure of tissue to repeated sequences of short ischemic periods (ischemic postconditioning) and reperfusion after a prolonged period of ischemia lessens ischemia-reperfusion injury via the activation of several anti-apoptotic mechanisms at the mitochondrial level (62). The clamping and unclamping of the spermatic cord may inflict injury to the testicular artery, which makes the technique unfit for the treatment of TIRI in humans. In addition, the clamping and unclamping of the vessel done in ischemic postconditioning cannot mimic the real clinical case of TT onset in humans, as testicular torsion is presented as a twisted spermatic cord and not as a result of clamping. Despite the shortcomings of both protocols in the treatment of TIRI in humans, experimental studies have shown that ischemic postconditioning is more protective effect than preconditioning because it does not only prevent free radicals' production, but also activates the activities of endogenous antioxidants and anti-apoptotic proteins to prevent oxidative damage and apoptosis, respectively (76). Ischemic postconditioning also activates reperfusion-injury rescue kinase (RISK) and JAK-STAT pathways as defense mechanisms to lessen ischemia-reperfusion injury (77). Nevertheless, increased mobilization and activation of the RISK or JAK kinases in prolonged testicular ischemia is considered injurious as it may induce tissue hypertrophy (78).

Finally, the pretreatment strategy for the management of ischemia-reperfusion injury (IRI) involves the administration of pharmacological agents before exposure to the periods of ischemia (79). Similar to ischemic preconditioning, pretreatment is done to increase tissue resilience to IRI (80). This includes the use of agents like cysteine, vitamin C and E, xanthine oxidase inhibitors (febuxostat), calcium channel blockers (amlodipine) and so on to minimize oxidative stress, inflammation and apoptosis. With a pretreatment strategy, several experimental studies on TIRI have reported lessened injury after the repair of induced testicular torsion (81). Similar to ischemic preconditioning and postconditioning, alleviation of testicular ischemia-reperfusion injury (TIRI) with a pre-treatment strategy is assumed to be unrealistic in clinical settings due to the unpredictability in the occurrence of testicular torsion in humans (82). However, some research has shown that application of pretreatment and ischemia preconditioning strategies may serve as prophylactic measures in conditions where IRI can be predicted, especially during organ transplant (83, 84).

## 5 Superiority of pharmacological postconditioning over other recommended treatment strategies

Pharmacological post-conditioning has great potential to be an effective approach in minimizing the harmful effects of ischemia-reperfusion (I/R) injury in testicular tissue (85). Protocols like ischemic preconditioning, postconditioning and pretreatments have been explored to alleviate ischemia-reperfusion injury, yet each approach has specific limitations (86). Ischemic preconditioning involves the induction of ischemia before the real ischemic event, making it unsuitable for sudden incidents, and its protective effects are often short-lived (87). Its efficacy also varies across tissues, and in some cases, it may increase damage, especially in patients with inflammatory or immune conditions (88). Postconditioning, meanwhile, must be applied immediately after reperfusion, limiting its use in emergencies, and its impact can vary based on patient-specific factors (89). Both approaches require precise timing and may benefit from a combination with other treatments for optimal results.

In recent years, there has been considerable research aimed at identifying effective strategies and pharmacological treatments to reduce or prevent testicular I/R injury. However, aside from scrotal cooling, no other methods have successfully transitioned into clinical practice. While the exact pathophysiological mechanisms behind testicular I/R injury are not fully understood, it is clear that the ROS generated during this process play a significant role. I/R injury has been shown to produce various toxic substances in the microcirculation of different tissues, along with potential damage to vascular endothelial cells and microcirculation disorders during reperfusion, leading to organ dysfunction (90).

Following ischemia-reperfusion (I/R) injury, the physiological environment undergoes significant changes, characterized by a halt in aerobic metabolism, metabolic acidosis, mitochondrial dysfunction, intracellular calcium overload, and the generation of reactive oxygen species (ROS) at the onset of reperfusion (83). Specifically, reperfusion injury leads to anoxia, which results in the excessive production of ROS, pro-inflammatory cytokines, cell adhesion molecules, and lipid peroxidation. This cascade activates necrosis and apoptosis pathways, causing further severe damage to ischemic tissues. The initial surge of ROS and the infiltration of pro-inflammatory neutrophils during the early stages of reperfusion are critical to the development of post-ischemic injury, highlighting the importance of early intervention to mitigate I/R damage (83).

In pharmacological postconditioning, drugs target pathways associated with reperfusion injury, such as oxidative stress and inflammation, to protect cells and promote tissue repair. Unlike preemptive treatments, it can be applied after the ischemic event, making it suitable for emergencies. This strategy has shown promise in reducing damage and improving recovery across various organs, including the heart, brain, kidneys, and testes (62, 91).

Unlike preconditioning, pharmacological postconditioning can be initiated after the ischemic event, making it applicable in emergencies where testicular torsion has already occurred (92). Additionally, pharmacological agents can be chosen to specifically counteract the pathways activated during reperfusion, such as inflammation, oxidative stress, and apoptosis (93). This targeted intervention allows for reduced damage to testicular tissue and fewer side effects compared to broader strategies. By directly influencing molecular damage mechanisms at the injury site, pharmacological postconditioning could support the preservation of fertility, hormone balance, and testicular structure in cases of testicular I/R injury (94). Its flexibility in application, potential for integration with other therapies, and tissue-specific action make pharmacological post-conditioning a valuable tool in clinical efforts to protect testicular health following ischemic events. Ongoing research is focused on refining drug selection, dosing, and timing to establish pharmacological post-conditioning as a superior approach for managing testicular ischemia-reperfusion injury and improving patient outcomes.

Although pharmacological postconditioning has shown substantial promise in experimental studies, claims of its superiority over ischemic conditioning protocols remain largely theoretical and based on preclinical findings. Most available data are derived from rodent or small-animal models in which drugs such as antioxidants, anti-inflammatory agents, phosphodiesterase inhibitors, and mitochondrial stabilizers demonstrated improved histological and biochemical recovery following testicular I/R injury (62, 85, 91). However, these findings are yet to be consistently validated in large-animal or human studies. The diversity of experimental models, differences in ischemia duration, reperfusion time, and drug pharmacokinetics can contribute to inconsistent outcomes across studies (93). Furthermore, almost no studies have directly compared pharmacological postconditioning to ischemic pre- or postconditioning under standardized conditions, making it difficult to establish clear superiority.

**Figure 3:**
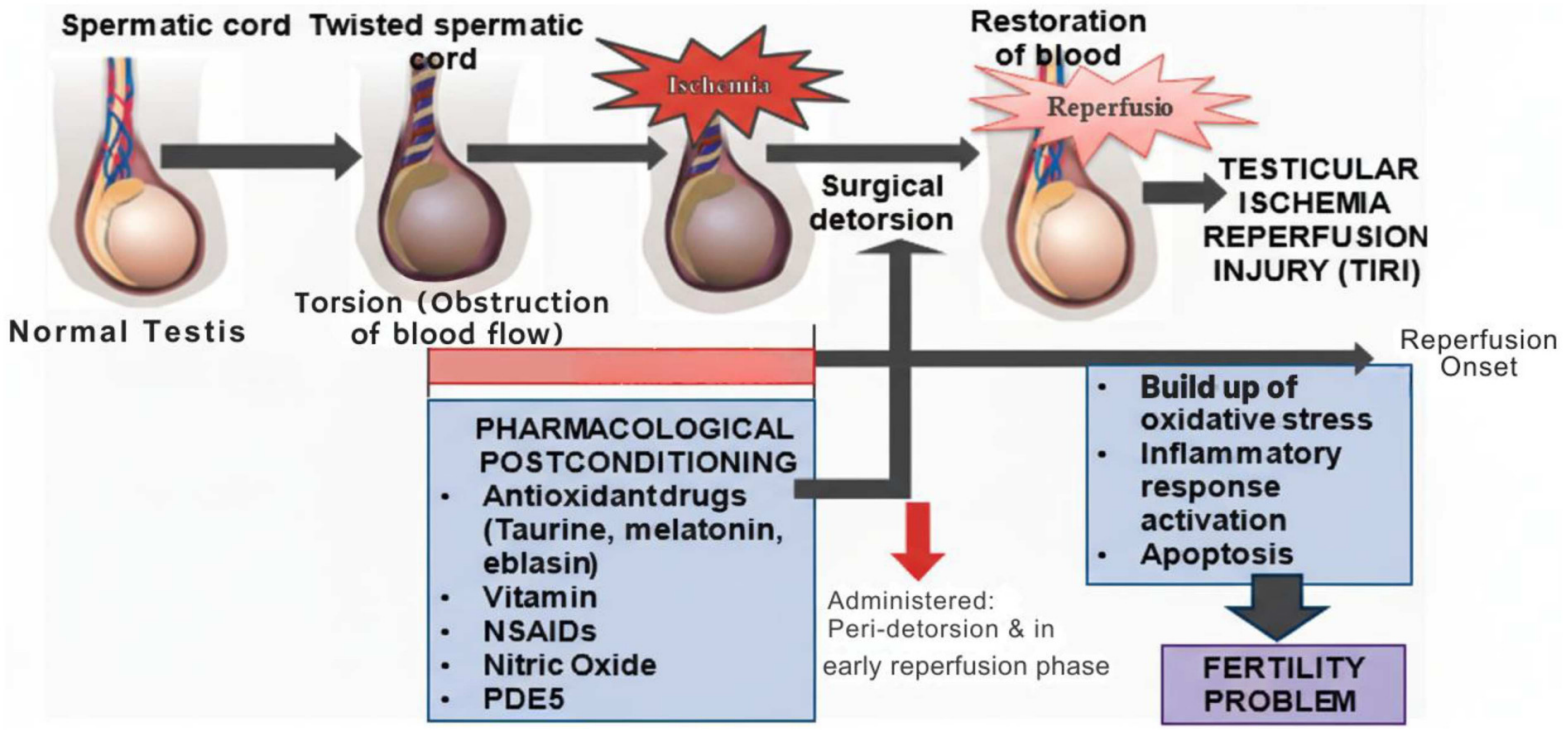
Application of pharmacological postconditioning on testicular torsion and detorsion.

Turner et al. (150) previously indicated that excessive free radical production occurs during testicular detorsion, suggesting that testicular torsion and detorsion represent classic examples of I/R injury. The mammalian testis is especially sensitive to oxidative damage from free radicals, making it essential to prevent the oxidative stress caused by the sudden influx of free radicals. This approach is crucial for minimizing damage to the ischemic area and reducing the likelihood of serious consequences, such as decreased fertility. Various pharmacological agents have been suggested and researched for their potential therapeutic applications in TIRI cases (Figure 3), some of which are discussed below.

### 5.1 Anti-oxidant drugs and free radical scavengers

Decreasing oxidative stress resulting from ischemia-reperfusion (I/R) is a critical target for pharmacological intervention, leading to investigations into various potential pharmaceutical agents, including Taurine, edaravone, melatonin, and apocynin (18, 95). For instance, Bilommi et al. (96) demonstrated that adult rats subjected to 4 h of ischemia followed by 3 h of reperfusion and treated with exogenous glutathione at the onset of reperfusion exhibited significantly lower levels of malondialdehyde (MDA) and reduced histopathological damage.

### 5.2 Taurine

Taurine, an organic acid found in mammalian tissues known for its antioxidant properties, has been utilized in models of testicular I/R. It has been shown to positively impact the ischemic testis by significantly reducing histopathological damage, apoptosis, and MDA levels, as well as markers for neutrophils and myeloperoxidase (MPO), and reversing damage to spermatogenesis caused by Ischemia-reperfusion injury (97, 98).

### 5.3 Edaravone

Tamamura et al. (99) created a short-term rat model of testicular I/R, where animals experienced unilateral torsion for 30 min followed by 1 h of reperfusion. In this study, a high dose of the free radical scavenger, edaravone, significantly decreased MPO levels, lowered HSP-70 protein activity, and partially reduced levels of NO_2_-NO_3_, MDA, and 8-hydroxy-2′-deoxyguanosine (a marker of oxidative DNA damage). It also diminished histological changes such as vacuolation and necrosis.

### 5.4 Melatonin

Melatonin, a potent antioxidant present in various tissues, has the potential to protect against I/R injury in the testes (100). According to Kurcer et al. (101) melatonin treatment significantly improved spermatogenesis and reduced histopathological damage, as well as lowered lipid and protein oxidation, indicated by decreased levels of MDA and MPO, along with reduced protein carbonyl groups. Kanter highlighted melatonin's protective effects against germ cell-specific apoptosis due to I/R and its ability to normalize proliferating cell nuclear antigen and testosterone levels (102). Additionally, melatonin treatment improved bilateral sperm concentration and positively affected sperm morphology.

### 5.5 Ebselen

Ebselen, a synthetic antioxidant that mimics the properties of glutathione peroxidase (GSH-Px), reacts with peroxynitrite and inhibits several enzymes, including lipoxygenase and nitric oxide synthase (103). Rifaioglu et al. (104) induced torsion-detorsion in adult rats for 2 h each and showed that ebselen had beneficial effects in reducing histopathological damage and enhancing spermatogenesis while increasing MDA and nitric oxide (NO) levels, suggesting its capability to scavenge reactive oxygen species.

### 5.6 Alpha-lipoic acid

Alpha-lipoic acid, important for mitochondrial dehydrogenase reactions, was studied by Ozbal et al. (105) who treated rats undergoing 2 h of ischemia and 2 h of reperfusion with alpha-lipoic acid administered 30 min before detorsion. Their results indicated the positive effects of alpha-lipoic acid on the activities of GSH-Px and superoxide dismutase (SOD), as well as reductions in MDA levels, histopathological damage, germ cell apoptosis, and caspase-3 detection via immunohistochemistry.

### 5.7 Simvastatin

Simvastatin, widely known as a lipid-lowering drug, also possesses significant antioxidant properties. Yang et al. (61) developed a model involving 4 h of testicular torsion followed by 24 h of detorsion, administering simvastatin at the onset of reperfusion. This treatment led to a significant reduction in bilateral histopathological damage, decreased MPO activity, and lowered levels of NO and MDA, as well as concentrations of pro-inflammatory cytokines TNF-α, IL-1β, and IL-6, and protein expression of NF-κB in both testes. The mechanisms behind these beneficial effects likely involve reducing NF-κB activation and mitigating I/R-induced oxidative stress.

Other antioxidants, such as resveratrol and apocynin, have also shown promising results in alleviating I/R-induced testicular damage. Yulug et al. (106) found that administering resveratrol intraperitoneally 30 min before reperfusion in a model of 4-hour ischemia followed by 24-hour reperfusion significantly reduced oxidative stress and total oxidative status, as well as decreasing histopathological damage and apoptosis in the torsion-detorsion group. Apocynin, extracted from the roots of the plant *Apocynum cannabinum*, inhibits nicotinamide adenine dinucleotide phosphate oxidase and has been shown to enhance the antioxidant enzyme system, promote reductions in glutathione, and regulate ischemia-induced cellular stress. Apocynin exerts its protective effects against I/R-induced oxidative damage by scavenging free radicals and boosting the antioxidant defenses of testicular tissue (107).

### 5.8 Vitamin E

Endogenous vitamins are vital for the antioxidant protection of various organs, including the testes (108). Vitamin E has been used in treating testicular torsion detorsion. Studies conducted by Romeo et al. (109) and Ranade et al. (110) indicated that administering a hydrophilic vitamin E-like antioxidant, raxofelast, 15 min before and after reperfusion in a model involving 3 h of ischemia followed by 4 h of reperfusion improved the structure of seminiferous tubules and reduced edema and hemorrhage in the affected testis. Furthermore, raxofelast significantly decreased levels of conjugated dienes, which are markers of lipid peroxidation, in both testes, suggesting its potential as a therapeutic agent for alleviating I/R-induced oxidative damage in the testes (111).

### 5.9 Ascorbic acid

Ascorbic acid has also been evaluated for its efficacy in treating testicular torsion–detorsion in comparison with dopamine. Research demonstrated that ascorbic acid was more effective than the vasodilator dopamine, leading to significant restoration of spermatogenesis and seminiferous tubule diameter, as well as a notable reduction in serum MDA levels. These results highlight the benefits of addressing oxidative stress to facilitate recovery in testes affected by I/R injury (112).

Considering the involvement of pro-inflammatory cytokines in testicular torsion–detorsion, some researchers have investigated the effects of anti-inflammatory drugs. Lysiak et al. (48) pointed out the importance of neutrophil recruitment to subtunical venules in germ cell-specific apoptosis linked to testicular I/R injury. In a study conducted in 2011, the neutrophil elastase inhibitor sivelestat in a protocol that involved 90 min of ischemia followed by 48 h of reperfusion in rats was employed. The findings revealed that sivelestat significantly reduced lipid peroxidation, germ cell-specific apoptosis, vacuolation, and necrosis in both the ischemic and contralateral testes (113).

### 5.10 Epigallocatechin-3-gallate

Epigallocatechin-3-gallate, a polyphenol found in green tea, has demonstrated the ability to inhibit inflammatory processes associated with carcinogenesis and reduce cell proliferation and oxidative stress (114). In a model involving 1 h of testicular torsion followed by 4 h of detorsion, epigallocatechin-3-gallate was administered intraperitoneally 30 min after ischemia. This treatment effectively reversed histopathological alterations and the decline in spermatogenesis, inhibited germ cell apoptosis, normalized pro-apoptotic gene expression (such as p53 and Bax), and reduced mRNA expression of inflammatory markers like inducible nitric oxide synthase and monocyte chemoattractant protein-1.

### 5.11 Interleukin-10 (IL-10)

Interleukin-10 (IL-10) is a cytokine known for regulating inflammatory responses by suppressing pro-inflammatory cytokines such as TNF-α, IL-1, IL-6, and IL-8 (115). Ozturk et al. (116) developed a rat model of 6 h of ischemia and 1 h of reperfusion, administering IL-10 intraperitoneally 10 min prior to reperfusion. Their results indicated that IL-10 treatment significantly lowered MDA levels, MPO activity, and histological damage while increasing GSH-Px activity. This study underscored IL-10's protective effects against testicular damage and its direct influence on seminiferous tubules.

### 5.12 Non-steroidal anti-inflammatory drugs (NSAIDs)

NSAIDs are commonly utilized to treat inflammation-related conditions, primarily by inhibiting cyclooxygenase, the key enzyme involved in the inflammatory process (117). Research by Dokmeci et al. (118) demonstrated that administering ibuprofen orally to prepubertal rats 40 min before the end of a 5-hour ischemia model resulted in significantly reduced levels of malondialdehyde (MDA) bilaterally and decreased endothelial nitric oxide synthase (eNOS) immunoreactivity in the contralateral testis, while the ipsilateral testis exhibited heightened eNOS immunoreactivity. Additionally, ibuprofen showed protective effects against mitochondrial degeneration in both Sertoli cells and spermatids.

Dexketoprofen, an active enantiomer of ketoprofen within the NSAID category, was tested in a 5-hour testicular ischemia model. It was administered intraperitoneally twice 40 min prior to reperfusion and again 12 h afterward. By 24 h post-reperfusion, dexketoprofen significantly lowered lipid peroxidation levels in both testes and mitigated degeneration, necrosis, disorganization, and desquamation of testicular tissue compared to control animals (119).

### 5.13 Nitric oxide (NO)

In a study, a short model involving 30 min of ischemia followed by 30 min of reperfusion to examine nitric oxide (NO) levels relative to blood flow. Results indicated that while blood flow plummeted to 5%–10% of normal after ischemia onset, NO levels gradually increased, peaking at the end of ischemia. Upon reperfusion, blood flow returned to normal within 5 min, but NO concentrations continued to rise. Additionally, administration of the NO donor, l-arginine, elevated NO_2_-NO_3_ concentrations and decreased MDA levels in a rat testicular ischemia-reperfusion model. Increased eNOS expression was also observed in the testis following torsion and detorsion, suggesting that NO exerts cytoprotective effects during ischemia-reperfusion events (120).

### 5.14 Phosphodiesterase type 5 (PDE5) inhibitors

Phosphodiesterase type 5 (PDE5) inhibitors, widely recognized for treating erectile dysfunction, pulmonary hypertension, and benign prostatic hyperplasia, have also demonstrated protective effects against ischemia-reperfusion injury in the heart. They do this by opening mitochondrial potassium channels and inhibiting calcium influx, thereby reducing cell death rates (121). Following the introduction of sildenafil citrate, several new PDE5 inhibitors have been evaluated for their effects in testicular ischemia-reperfusion models. The results have generally indicated that sildenafil, tadalafil, and udenafil offer protection against oxidative damage in the testis, although findings regarding vardenafil have been mixed. In a study involving testicular torsion (2 h) followed by detorsion (8 h) in pigs, vardenafil did not show any beneficial effects and instead worsened histopathological changes related to oxidative stress (122). Conversely, in a rat model of testicular torsion (1 h) followed by detorsion (4 h), vardenafil administered 30 min after ischemia induction reduced levels of apoptosis-related factors, eNOS, and inducible nitric oxide synthase, ultimately alleviating cellular damage and suggesting a potential cytoprotective effect.

Sildenafil citrate has consistently demonstrated protective effects against oxidative stress and histological alterations across various studies. Tadalafil has recently shown promise in preserving antioxidant capacity, as evidenced by increased superoxide dismutase (SOD) levels and decreased MDA content in a rat model (123). Finally, research by Özgür et al. (124) indicated that udenafil significantly improved biochemical changes when administered before reperfusion in a rat model of testicular torsion (2 h) followed by detorsion (4 h), reducing edema and hemorrhage and restoring germ cell organization in the seminiferous tubules. These PDE5 inhibitors seem to exert their positive effects by mitigating oxidative stress and enhancing the tissue's antioxidant capacity. Future studies may uncover more details about the mechanisms of action for PDE5 inhibitors, further elucidating their potential as therapeutic agents.

The timeline illustrates the progression from normal testis to torsion-induced ischemia, followed by surgical detorsion and reperfusion. Drug administration for pharmacological postconditioning is timed around the detorsion phase, aiming to mitigate reperfusion-mediated oxidative stress, inflammatory activation, and apoptosis. The use of antioxidant drugs (taurine, melatonin, eblasin), vitamins, NSAIDs, nitric oxide donors, and PDE5 inhibitors immediately before or after reperfusion may improve testicular salvage and reduce fertility impairment.

## 6 Pharmacological applications of febuxostat, amlodipine and vitamin E against testicular ischemia-reperfusion injury

Based on the multi-factorial nature of the TIRI pathway (20), pharmacological postconditioning may help to limit testicular damage by blocking key points on the TIRI pathways through the use of different drugs. In addition to this, pharmacological postconditioning helps to enhance treatment options that can be applied to human subjects seeking medical help after testicular torsion onset and repair, unlike other treatment regimens that cannot be applied for the treatment of TIRI in humans in real-life situations. From careful investigation of TIRI pathways, events that contributed to testicular damage occur sequentially or in a specific pattern. Firstly, there is a build-up of xanthine oxidase (XO) enzymes in the ischemic phase of testicular torsion, which activates reactive oxygen species that can inflict injury on testicular cells within a short period or form complexes with other radicals to form peroxynitrites that can cause long-term damage to the testes (151, 152). This event may serve as a basis for tissue damage during reperfusion. For this reason, blocking the XO-ROS pathway in the ischemic phase of testicular torsion (TT) could serve as a means of reducing testicular damage that may exacerbate testicular damage during reperfusion (60). Blocking this pathway should be done immediately after testicular torsion is diagnosed to limit XO-ROS-driven testicular damage during reperfusion.

From previous studies, febuxostat, a xanthine oxidase (XO) inhibitor has been reported to exhibit a superior antioxidant protection over other XO inhibitors (153). It has been reported to block the reduced and oxidised form of xanthine oxidase. Febuxostat has been documented to exhibit antioxidant, anti-inflammatory, anti-apoptotic and cytoprotective properties (41) (153, 154). It has been previously used to attenuate myocardial and renal ischemia-reperfusion injury (155–157). It is therefore speculated that administration of febuxostat a few minutes before surgical detorsion may limit XO-driven ROS production that may inflict injury on the testes during reperfusion.

In addition to this, another event during reperfusion is calcium overload due to disruption of the sodium-calcium ATPase pump (45). This may result in activation of the calcium-mediated apoptotic pathway, which may result in loss of testicular cells. Also, there is altered vasomotor tone after testicular torsion repair, which does not return to normal even after 7 days, and may disrupt the spermatogenesis (158). To impede this pathway, amlodipine, a third-generation calcium channel blocker, is capable of blocking the lipid peroxidation process (159, 160). It has been reported to be effective in minimizing vascular-induced damage in organs (161). It has been reported to protect against TIRI and other forms of ischemia-reperfusion injury (162, 163). Administration of amlodipine immediately after TT repair may help to limit the calcium-mediated apoptotic pathway and regulate the loss of vasomotor tone. The final sequence of events following TIRI is the activation of ROS-burst in the later phase of reperfusion due to leukocyte recruitment to the site of injury (50). This event occurs hours after testicular torsion repair and increases the intra-testicular ROS, which tends to trigger apoptotic pathways (32). Vitamin E, a lipid-chain-breaking antioxidant known for its ability to improve reproductive function, may help to block this event. Administration of vitamin E 30 min after reperfusion may help to limit the ROS-burst in the later phase of reperfusion that may inflict injury on the testicular cells.

It is important to note that while these pharmacological candidates demonstrate protective potential in preclinical models, their translational applicability to human testicular ischemia-reperfusion injury remains uncertain. Most reports supporting the use of febuxostat, amlodipine, and vitamin E derive from rodent studies employing experimental ischemia protocols and dosages that do not necessarily reflect safe or effective human exposure levels (60, 85, 125, 163). Pharmacokinetic variables, including drug bioavailability in testicular tissue, blood–testis barrier permeability, and metabolism under ischemic conditions, are often poorly characterized. For example, the optimal timing and systemic concentration of febuxostat required to inhibit xanthine oxidase in the human testis remain unknown, and excessive dosing may impair purine metabolism or hepatic function (154, 155). Similarly, while amlodipine exhibits antioxidative and vasomodulatory effects in animals, its testicular tissue penetration and potential influence on systemic blood pressure in acute torsion cases require further clinical evaluation.

At present, there are no controlled clinical trials or pharmacodynamic studies assessing these drugs specifically in patients with testicular torsion or related ischemia-reperfusion contexts. Therefore, while preclinical findings support their mechanistic potential, claims of therapeutic superiority or clinical readiness remain premature. Future translational research should focus on establishing safe dosing windows, evaluating pharmacokinetic behaviour in gonadal tissue, and integrating these agents into combinatory or adjunctive regimens under standardized experimental and clinical frameworks.

However, targeting this series of events with febuxostat, amlodipine, and vitamin E sequentially may still help to limit testicular damage following TIRI.

## 7 Conclusion

This review highlighted that TIRI is accompanied by cascades of pathophysiological events that cause testicular damage. With the application of pharmacological postconditioning, there is the possibility of blocking the TIRI pathways with multiple interventions (febuxostat, amlodipine and vitamin E) to limit events that contribute to testicular damage. This suggests that pharmacological postconditioning could serve as a treatment strategy that allows febuxostat, amlodipine and vitamin E to be administered in a sequence to reduce the risk of TIRI after TT onset and repair.

## 8 Recommendation, limitations, and contribution to knowledge

Based on the multifaceted nature of TIRI pathway, this review recommends administration of febuxostat in the ischemic phase to block XO-ROS pathway, administration of amlodipine on detorsion to block calcium-mediated apoptotic pathway and administration of vitamin E in the later phase of detorsion to reduce TIRI-induced testicular damage. This review recommends a pattern of application of therapeutic drugs that can be applied in humans to treat TIRI-induced testicular damage after TT repair.

It must, however, be noted that, while pharmacological postconditioning offers practical advantages, such as feasibility in unpredictable clinical events like testicular torsion and the potential for targeted molecular modulation, its current evidence base does not yet support definitive claims of clinical superiority. Most pharmacological agents remain in the exploratory stage, with uncertain safety profiles, optimal timing, and long-term fertility outcomes in humans. In contrast, ischemic conditioning strategies, despite logistical limitations, have undergone limited but more direct testing in clinical or surgical contexts, particularly in cardiac and renal reperfusion models. Therefore, a rigorous head-to-head evaluation of pharmacological vs. ischemic conditioning across comparable experimental frameworks, followed by controlled translational studies, is necessary before fully asserting pharmacological postconditioning as a superior therapeutic strategy for TIRI.
